# Emotion Controllability Beliefs and Young People’s Anxiety and Depression Symptoms: A Systematic Review

**DOI:** 10.1007/s40894-023-00213-z

**Published:** 2023-04-29

**Authors:** Matthew P. Somerville, Helen MacIntyre, Amy Harrison, Iris B. Mauss

**Affiliations:** 1 Department of Psychology and Human Development, UCL Institute of Education, London, UK; 2 Department of Psychology, University of California, Berkeley, USA

**Keywords:** Emotion controllabilitybeliefs, Emotion regulation, Anxiety, Depression, Adolescent mental health

## Abstract

Emotion regulation is a powerful predictor of youth mental health and a crucial ingredient of interventions. A growing body of evidence indicates that the beliefs individuals hold about the extent to which emotions are controllable (emotion controllability beliefs) influence both the degree and the ways in which they regulate emotions. A systematic review was conducted that investigated the associations between emotion controllability beliefs and youth anxiety and depression symptoms. The search identified 21 peer-reviewed publications that met the inclusion criteria. Believing that emotions are relatively controllable was associated with fewer anxiety and depression symptoms, in part because these beliefs were associated with more frequent use of adaptive emotion regulation strategies. These findings support theoretical models linking emotion controllability beliefs with anxiety and depression symptoms via emotion regulation strategies that target emotional experience, like reappraisal. Taken together, the review findings demonstrate that emotion controllability beliefs matter for youth mental health. Understanding emotion controllability beliefs is of prime importance for basic science and practice, as it will advance understanding of mental health and provide additional targets for managing symptoms of anxiety and depression in young people.

## Introduction

The association between emotion regulation and youth mental health, in particular depression and anxiety symptoms, is well established (Moltrecht et al., 2021; Schaefer et al., 2017). This work has raised important questions regarding what leads young people to engage in emotion regulation. A growing body of evidence indicates that the beliefs young people hold about the extent to which emotions are controllable (emotion controllability beliefs) influence both emotion regulation and subsequent mental health. This idea has critical implications for basic science and practice as it provides a better understanding of antecedents of emotion regulation and points to targets for intervention. Yet, no systematic review of this emergent body of research has been undertaken. This article provides such a review, focusing on the extent to which emotion controllability beliefs are associated with symptoms of youth anxiety and depression and the emotion regulation mechanisms accounting for these links.

Emerging research and recent theorizing suggest that emotion controllability beliefs play a major role in young people’s depression and anxiety symptoms (Crawford et al., 2021; De Castella et al., 2013; Ford & Gross 2019). Current theoretical models (Ford & Gross, 2019; Kneeland & Dovidio et al., 2016) propose that these beliefs influence anxiety and depression symptoms via emotion regulation strategies that target emotional experience, like cognitive reappraisal. Individuals who believe emotions are relatively controllable are more likely to attempt to regulate their emotions and to persist in these efforts. Conversely those who believe they have little control over their emotions will be less motivated to engage in emotion regulation and perhaps expend less effort in doing so. For example, when anxious about an upcoming test, one person may believe there is very little they can do about their anxiety symptoms, whereas another may believe they can—if they wish to—successfully decrease their anxiety symptoms as they head into the test. This person might, in turn, be more motivated to engage in emotion regulation and be more successful in doing so. After all, it only makes sense to attempt to exert control if one believes these attempts will be successful. In the words of Ford et al. (2018), this distinction is between beliefs that emotions can be shaped and modulated according to one’s will versus emotions arriving unbidden and departing of their own accord.

Emotion controllability beliefs tend to be normally distributed, from believing one has very little to a great deal of control over emotions, with most individuals falling in the middle (Becerra et al., 2020). They are thus conceptualized as a meaningful individual-difference variable. Importantly, these beliefs appear to not yet be fixed during adolescence (Ford et al., 2018), indicating this is a particularly impactful time to intervene, preventing a potential decline in beliefs about controllability before they become more fixed in adulthood.

The idea of examining people’s emotion controllability beliefs is rooted in Bandura’s (1997) work on self-efficacy and the work of Carol Dweck and colleagues on ‘self-theories’(Diener & Dweck, 1978; Dweck, 2006), which encompasses the fundamental beliefs individuals hold about whether intelligence can be controlled and changed. Subsequent research on beliefs about intelligence and, later, about emotion (Tamir et al., 2007) has used different labels for these beliefs, including self-theories, implicit theories, lay theories, and mindsets (King & dela Rosa, 2019; Schroder et al., 2015; Tamir et al., 2007). In this review, the term beliefs is used as it refers to a single idea about emotions rather than an organized set of beliefs that would constitute a theory or a mindset, and because it is not clear how implicit (versus explicit) these beliefs are. Similarly, existing research has used different labels for the dimension of controllability, including malleable (versus entitative) and growth or incremental (versus fixed). Here, the term controllability is used because it most concretely and precisely communicates the core of the belief: the extent to which emotions are controllable.

Within emotion controllability beliefs, a distinction can be made between beliefs about the controllability of emotions in general (a belief that ‘if they want to, people can change the emotions they have’; general emotion controllability belief) and a belief about one’s own, personal capacity to control one’s own emotions (a belief that ‘if I want to, I can change the emotions I have’; personal emotion controllability belief). The two might be similar in that general emotion controllability beliefs might inform and be informed by personal beliefs. At the same time, there might also be important differences in that, at least in principle, what one believes people in general can do with their emotions might diverge from what one believes oneself can do. For example, a person might think that people in general can control their emotions, but they personally have little capacity to do so. In turn, the two types of beliefs might have distinct implications for mental health. It is thus important to consider this distinction when examining emotion controllability beliefs. The literature also includes a small number of studies that have used second person statements in their measures (e.g., “You can learn to control your emotions”). Ford et al. (2018) note the ambiguity of measures where items are in this second person form, as it is unclear whether the respondent is referring to themselves or others.

Another potentially important distinction comes from the target of the emotion controllability belief. Specifically, just like people might hold different beliefs about emotion versus intelligence (Tamir et al., 2007) they might hold different beliefs about different emotional targets, including anxiety-related versus depression-related emotions. For example, someone might believe emotions are relatively controllable when thinking about anxiety but less controllable when thinking about sadness. Therefore, where available, research that has identified emotion controllability beliefs with discrete emotion targets has been considered.

## Current Study

Emotion regulation strategy and how it relates to youth mental health and well-being has been well documented. Recent theory and a growing body of evidence suggests that emotion controllability beliefs are a key antecedent of emotion regulation and are associated with symptoms of anxiety and depression in young people. Despite the basic-scientific and practical importance of these links, no systematic review of the scientific evidence base has been conducted. The current study systematically reviewed the literature on emotion controllability beliefs and symptoms of anxiety and depression in young people aged 14–24 years. The overarching research question was to what extent, and by what process are emotion controllability beliefs associated with symptoms of anxiety and depression in young people? Specifically, the review examined distinctions between personal and general beliefs, cognitive reappraisal and suppression, anxiety and depression, and the influence of age and gender.

## Methods

### Procedure

**Fig. 1:**
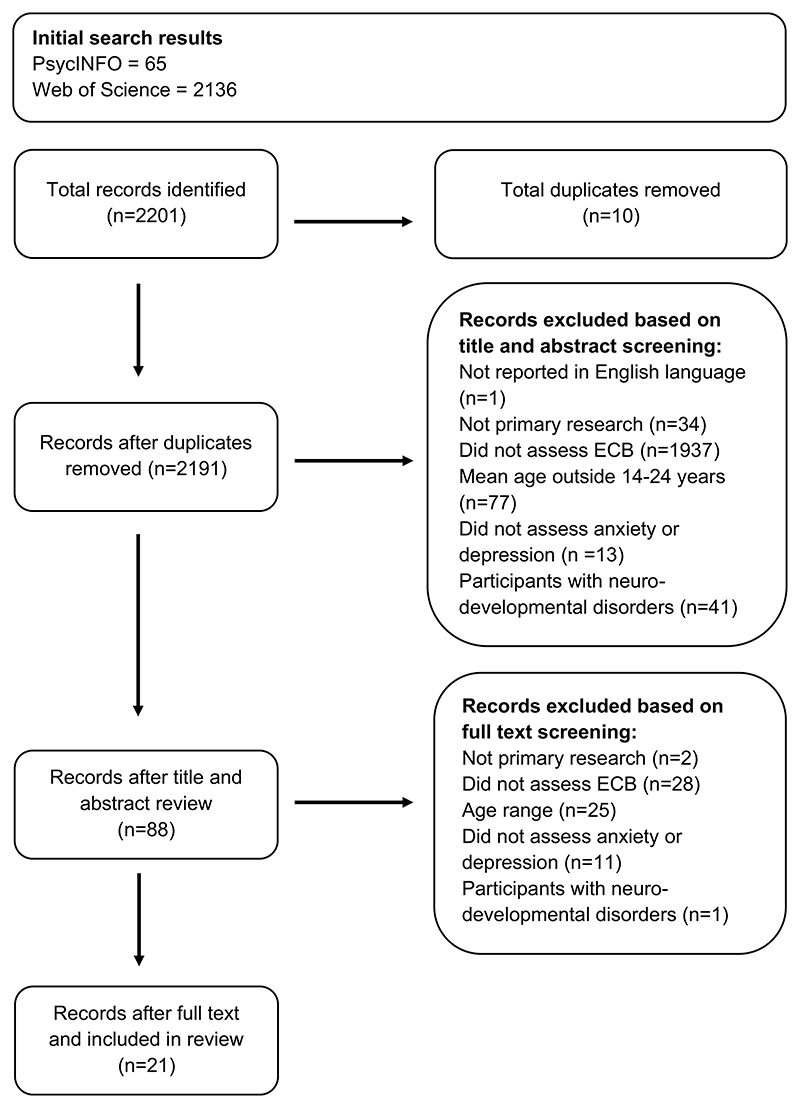
Flow diagram of the study selection process

A systematic review was conducted following guidance by Gough et al. (2017). The literature search was initially carried out in August 2021 and updated in February 2023, using Ovid PsycINFO and Web of Science. Table 1 provides a summary of the included studies (see https://osf.io/wb259/ for the full protocol, including search strategy) and Fig. 1 provides the flow diagram, illustrating the identification, screening, and inclusion processes. No studies were excluded based on year of dissemination.

All articles identified in the literature search were added to the EPPI Reviewer (Web) software. Duplicates were removed and the remaining articles were screened by MS and HM based on the inclusion and exclusion criteria detailed below. Inter-rater reliability regarding each article’s eligibility for inclusion was 96.8% for title and abstract screening and 91.3% for full text screening. Any disagreements were discussed until a consensus was reached. Further scrutiny and cross-checking were carried out with the remaining articles at both the title/abstract and full-text screening stages.

This review was co-designed to utilize input from a Youth Panel of 8 individuals with lived experience of depression and/or anxiety symptoms (*M*_age_ 19.25). Members were recruited via social media and were diverse in terms of location, with individuals from Bermuda, India, Nigeria, Rwanda, and the United Kingdom. The Youth Panel met three times throughout the review process. A Clinician Panel was also assembled that met twice and included three clinicians working with young people with symptoms of depression and anxiety. The panels helped inform the research team’s thinking in areas such as search terms, directions for future research, and dissemination of outputs for key stakeholder groups.

### Inclusion Criteria

The following study designs were included in the systematic search:

- Cross-sectional studies identifying associations between emotion controllability beliefs and symptoms of anxiety and/or depression.
- Longitudinal studies identifying associations over time between emotion controllability beliefs and symptoms of anxiety and/or depression.
- Experimental studies including interventions focused on emotion controllability beliefs and broader interventions which include a component targeting emotion controllability beliefs.
- Qualitative or mixed methods studies that examine emotion controllability beliefs and symptoms of anxiety and/or depression.

#### Participants

- We only included studies in which the participant mean age fell between 14 and 24 years.

#### Measures

- We included studies that examined emotion controllability beliefs AND one or more of the following: anxiety, depression, mental illness, mental health, psychological health, well-being.

#### Language of review

- We only reviewed studies reported in English. However, there were no restrictions regarding language of assessment or geographical location of studies.

## Exclusion Criteria

The following were excluded from the systematic search:

- Unpublished manuscripts.
- Encyclopedia entries.
- Conference abstracts and presentations.
- Book reviews and chapters.
- Letters and editorials.
- Review and conceptual articles.
- Studies recruiting individuals with neurodevelopmental conditions.

## Data Extraction

Information relating to the following study characteristics was collected: study aims, conceptualization of emotion controllability beliefs, context, geographic location, research design, participant characteristics, data collection methods, data analysis methods, findings, and stated implications.

### Assessment of Quality and Relevance of Study

Following data extraction, the quality of the evidence from the included studies was evaluated using Kmet et al.’s (2004) standard quality assessment criteria. For 20 of the 21 studies, the quantitative checklist was used. Three items from the original checklist were discarded, as they related to random allocation and blinding within intervention studies, which applied to only one of the included studies. This resulted in an 11-item checklist being used for the quantitative studies. For Criterion 2, *Is the study design evident and appropriate?* only studies using experimental or longitudinal designs were accorded the maximum score. For Criterion 3, *Is the Method of subject/comparison group selection or source of information/input variables described and appropriate?* those studies that used the ambiguous second person scales were given a maximum score of 1. The one qualitative study in the review was evaluated using Kmet et al.’s (2004) 10-item quality assessment criteria for qualitative studies.

For both the quantitative and qualitative checklists, items were scored as not meeting (0), partially meeting (1), or fully meeting (2) each criterion (see Tables 2 and 3). To calculate the overall quality score for each study, the scores were summed and divided by the highest possible score (22 for the quantitative checklist, 20 for the qualitative checklist). Studies were also given a relevance rating (low/medium/high), based on how useful the study was in addressing the overarching research question of the review.

## Data Synthesis

Studies were synthesized using a Framework Synthesis approach, as described in Gough et al. (2017). This approach involved developing an initial conceptual frame-work at the outset of the review process to organize studies into groups based on relevant dimensions associated with the overarching research question. It also allowed for further categories to emerge during the synthesis as the literature was reviewed.

## Results

### Quality and Relevance Ratings

Quality and relevance ratings for each study are presented in Tables 2 and 3. The quality score columns demonstrate all studies score higher than 0.75 indicating that all reviewed studies were judged to be of sufficient quality for the analysis.

There was more variability in the relevance rating given to each study which assessed how useful study findings were for addressing the research question for this review. High relevance studies were centrally focused on the association between emotion controllability beliefs and symptoms of anxiety/depression and gave substantial consideration to mechanisms linking them. Medium relevance studies had some focus on the association between anxiety/depression symptoms and gave some consideration to mechanisms. Low relevance studies had some focus on the association between emotion controllability beliefs and anxiety/depression symptoms. Ten of the studies were judged to be highly relevant to this review, 9 of medium relevance and 2 of low relevance.

### Emotion Controllability Beliefs and Symptoms of Anxiety

Cross-sectional and longitudinal studies found that believing emotions are relatively controllable was consistently associated with fewer symptoms of anxiety in young people. Of the 21 studies in the review, 11 assessed sympstoms of anxiety. Table 4 presents the key findings of these studies and is structured by the type of measure that was used to assess emotion controllability beliefs (general vs. personal vs. anxiety/depression specific).

Daniel et al. (2020) found that in a sample of young people with elevated social anxiety symptoms, those who believed emotions were relatively controllable had fewer symptoms of anxiety (*r* = − .27). King & dela Rosa (2019) found a similar pattern when using a second person measure of emotion controllability beliefs with university students, yet with a smaller (though still statistically significant), effect size (r = − .11). Conversely, a much larger effect size (*r* = − .74) was found between emotion controllability beliefs and anxiety symptoms in Russell et al.’s (2021) study of young people with cystic fibrosis. In the studies that used anxiety-specific measure of emotion controllability beliefs, similar patterns were found. Schroder et al.’s (2015) cross-sectional study found that their Theory of Anxiety (TOA) measure had consistently stronger correlations with all anxiety outcomes (*rs* = − 0.35 to − 0.44) than Tamir’s (2007) general emotion controllability beliefs measure (*rs* = − 0.09 to − 0.29). Zhu et al. (2021) used a similar anxiety-specific scale and found a strong negative correlation between beliefs that emotions are controllable and anxiety symptoms (*r* = − .*53*).

The current review also identified two longitudinal studies that examined emotion controllability beliefs and anxiety symptoms in young people. Schroder et al.’s (2019) study provides some evidence, at least in the short term, for the proposition that young people’s emotion controllability beliefs precede anxiety symptoms: they found that prior week’s ‘growth anxiety mindset’ contributed, beyond base-line ratings of depression symptoms, to subsequent distress (a composite of worry, anxiety, loneliness, depression, and anger items), an average of *−* 0.35 SD across the 5 weeks. Mesurado et al. (2018) tested three models in the opposite direction, which included (among other variables) anxiety symptoms at Wave 1 predicting three types of emotion controllability beliefs (positive affect, despondency/distress, and anger/irritation) at Wave 2. Anxiety symptoms did not predict emotion controllability beliefs in any of these models.

Taken together, these findings suggest that young people who believe emotions are relatively controllable tend to have fewer symptoms of anxiety. However, the majority of these findings were cross-sectional, and more research needs to be carried out examining these effects across different time points to gain a better understanding of the direction of this relationship.

### Emotion Controllability Beliefs and Symptoms of Depression

Young people who believe emotions are controllable seem to be less likely to develop symptoms of depression. Evidence for this comes from 18 studies in the review that assessed symptoms of depression. As with the anxiety studies, a range of different emotion controllability beliefs measures were used. There were four cross-sectional studies that found a negative association between general emotion controllability beliefs and depression symptoms. The majority found a weak to moderate effect size (r = − .12 to r = − .28; De Castella et al., 2013; Ford et al., 2018; King & dela Rosa, 2019; Skymba et al., 2020), with the exception of Skymba et al.’s (2020) Study 2 which found a strong association between emotion controllability beliefs and depression symptoms for the 14–18-year-old group (*r* = − .52). The effect sizes in studies that used a personal emotion controllability beliefs measure were also mostly in the weak to moderate range (r = − .15 to r = − .45; Caprara et al., 2008, 2010; Daniel et al., 2020; De Castella et al., 2013; Di Giunta et al., 2022; Mesurado et al., 2018). However, in Russell’s (2021) study looking at emotion controllability beliefs in young people with cystic fibrosis, their novel measure of personal emotion controllability beliefs was strongly negatively correlated with depression symptoms (*r* = − .78). Additionally, Zhu et al. (2021) used a depression-specific measure of emotion controllability beliefs and found moderate to strong correlations with depression symptoms in two different analyses (*r* = − .47 and *r* = − .50).

There is also longitudinal evidence supporting the link between emotion controllability beliefs and depression symptoms, providing insight into the direction of this relationship. Five of the reviewed studies examining depression symptoms used a longitudinal design. In the first study to use the ITES measure, Tamir et al. (2007) found that believing emotions were controllable before starting university was associated with fewer symptoms of depression at the end of the students’ first year of undergraduate study (*r* = − .14). However, they did not control for baseline depression symptoms, making it difficult to determine whether emotion controllability beliefs predicted change in symptoms of depression. Schroder et al. (2019) also found associations between anxiety-focused emotion controllability beliefs and symptoms of depression across a five-week period, while Caprara et al. (2010) found links between personal emotion controllability beliefs and depression symptoms up to four years later (*r* = − .27). Some of the reviewed studies tested models in the opposite direction, examining whether depression symptoms predicted emotion controllability beliefs. Mesurado et al. (2018) tested three different models, each using a different measure of emotion controllability beliefs and found that depression symptoms at Time 1 did not predict emotion controllability beliefs relating to despondency or anger but did predict emotion controllability beliefs relating to positive emotions at Time 2. Crawford et al. (2021) also tested both directions and found that emotion controllability beliefs predicted depression symptoms 18 months later, but depression symptoms did not predict subsequent emotion controllability beliefs.

In the only qualitative study of the review, the secondary school students who were interviewed following a class-room-based universal prevention program for depressive symptoms reported that they did not believe they could control negative emotions using strategies such as reappraisal or mindful breathing. Instead the participants argued that avoidance strategies were more effective and believed that more cognitively engaged strategies, such as reappraisal, were only effective at a later stage, when the intensity of the negative emotion has reduced (Tang et al., 2022).

While the longitudinal studies offer support for the view that emotion controllability beliefs exert a causal influence on both anxiety and depression symptoms, further evidence is needed. One way of testing these causal hypotheses more directly is through experimentally manipulating these beliefs and measuring the effects of such manipulations on mental health outcomes.

### Emotion Controllability Beliefs and Experimental Manipulation Studies

Of the 21 studies that met the inclusion criteria, only one used an experimental design. In this study, Kneeland et al. (2016) randomly allocated participants from a community sample (*M*_age_ = 21.6, *SD* = 4.19) to an ‘emotions are controllable’ (n = 41) or ‘emotions are uncontrollable’ condition (n = 48). The experimental manipulation involved participants reading a one-page passage which presented fictitious data and quotations to convey the argument that emotions were either controllable or uncontrollable. Following this, all participants were asked to complete an impromptu, brief speech task that was designed to elicit symptoms of anxiety. As predicted by the authors, those who were induced to see their emotions as being controllable were more likely to use cognitive reappraisal to regulate their anxiety symptoms during the speech task. However, no link was found between this increased use of reappraisal and anxiety symptoms.

### What Processes Link Emotion Controllability Beliefs and Anxiety and Depression Symptoms?

Theoretical models posit that the more one believes emotions are controllable, the more likely they are to take steps to regulate their emotions, leading to reductions in symptoms of anxiety and depression (Ford & Gross, 2019). This section examines the indirect effect of emotion controllability beliefs on anxiety and depression symptoms in young people via emotion regulation and related pathways.

A number of studies in this review examined the associations between emotion controllability beliefs and cognitive reappraisal and/or expressive suppression (Daniel et al., 2020; De Castella et al., 2013; Ford et al., 2018; King & dela Rosa, 2019; Schroder et al., 2015; Tamir et al., 2007). In all of these studies, a significant positive association was found between emotion controllability beliefs and cognitive reappraisal (Personal emotion controllability beliefs *rs* = − 0.24 to − 0.46; General emotion controllability beliefs *rs* = − 0.17 to − 0.35). In contrast, suppression was only significantly associated with emotion controllability beliefs in one study out of the three that examined this link. Schroder et al. (2015) found weak associations between anxiety specific emotion controllability beliefs and suppression in two analyses (*rs* = − 0.13 to − 0.23).

Links between emotion regulation strategy use and anxiety or depression symptoms have been well established (Aldao et al., 2010), however, only three of the reviewed studies specifically tested mediation models that examine the effects of emotion controllability beliefs on anxiety and depression symptoms, via emotion regulation. Ford at al.’s (2018) findings were in line with hypothesized models showing that general beliefs that emotions are controllable predicted fewer depressive symptoms, via increased cognitive reappraisal (but not expressive suppression). De Castella et al. (2013) found a similar effect with cognitive reappraisal mediating the association between personal emotion controllability beliefs and depression symptoms. In contrast, King and dela Rosa (2019) found that reappraisal only mediated the link between general emotion controllability beliefs and positive emotions and not with anxiety and depression symptoms. This study used a shortened 3-item version of a commonly used measure of cognitive reappraisal (ERQ; Gross & John 2003). Internal consistency was not reported for this adapted scale, and it is possible this may explain this difference. However, it could also relate to this study being carried out in the Philippines, one of the few studies conducted in a non-Western context.

There were also studies that went beyond these two strategies when examining potential mediators in the link between emotion controllability beliefs and mental health. Caprara et al. (2010) investigated the role of interpersonal variables in the association between emotion controllability beliefs and mental health outcomes. They found that both *filial self-efficacy* (self-efficacy for handling emotions during interactions with parents) and *empathic self-efficacy* (self-efficacy for sensing and responding to someone else’s emotional needs during interactions) mediated the relationship between personal emotion controllability beliefs and depression symptoms. Believing that emotions were relatively controllable predicted higher levels of both types of self-efficacy which in turn predicted fewer symptoms of depression.

Skymba et al. (2020) extended the emotion regulation findings further by examining conceptually related groups of emotion regulation and emotion dysregulation measures. They found that both *disengagement* (e.g., cognitive avoidance) and *emotion dysregulation* (e.g., rumination) mediated the link between general emotion controllability beliefs and depression symptoms. Specifically, believing that emotions are controllable led to lower levels of disengagement and dysregulation and fewer symptoms of depression.

### Age and Emotion Controllability Beliefs

As can be seen in Table 1, the current review covers a broad range of ages (14–24). Twelve of the 21 studies targeted university students, nine studies targeted high school students, three studies examined middle school students, and two studies targeted primary school students. Due to the number of different measures used across the studies, it is difficult to compare some of the studies. However, mean scores on the most widely used scale, *Implicit Theories of Emotion Scale* (Tamir et al., 2007) indicate that across the studies, younger participants were more likely to believe that emotions are controllable than older participants.

While few of the reviewed studies examined emotion controllability beliefs and age specifically, of those that did, a similar trend was observed. Crawford et al. (2021) had the largest age range of young people (7- to 18-year-olds) and found that the general belief that emotions were controllable was negatively correlated with age (r = *−* .23). Ford et al. (2018) carried out three separate studies with different participant groups, and although their first study of 14–18-year-olds did not find a link between age and general emotion controllability beliefs, their second study, which looked at a younger group of participants across a larger age range (aged 8–16 years) over a period of 18 months, found that participants’ beliefs that emotions are controllable decreased across the period of the study. Their data also indicated that emotion controllability beliefs may be more closely linked with puberty than age. This was based on an analysis comparing young people who were of different ages and had the same pubertal status (i.e., prepubertal 4th vs. 7th graders; pubertal 7th vs. 10th graders) and young people who were the same age but had a different pubertal status (i.e., prepubertal 7th graders vs. pubertal 7th graders). Furthermore, based on comparisons with adult participant scores, their data suggest that beliefs appear to remain relatively stable after 17–18 years.

### Gender and Emotion Controllability Beliefs

Four of the general emotion controllability beliefs studies addressed gender differences in emotion controllability beliefs. King and dela Rosa (2019), who included a baseline measure of ‘trait malleability beliefs’ in their experimental study, found no gender difference. Tamir et al. (2007) found no consistent relationship between gender and emotion controllability beliefs. However, Ford et al. (2018) and Crawford et al. (2021) both found that boys believed emotions to be more controllable than girls. Neither study found a moderating effect of gender on the association between emotion controllability beliefs and symptoms of depression.

Skymba et al. (2020) examined a wider range of emotion regulation mediators between general emotion controllability beliefs and depression symptoms and found indications of pathway differences. Specifically, in their study of 14-18-year-olds, the negative association between believing emotions are relatively controllable and emotion disengagement was stronger for boys. They also found a stronger negative association between believing emotions are relatively controllable and emotion dysregulation for girls, and the indirect effect of emotion controllability beliefs on depressive symptoms, via emotion dysregulation was significant for girls but not boys.

In terms of personal emotion controllability beliefs studies, findings showed no gender differences in these beliefs (Daniel et al., 2020; De Castella et al., 2013). However, there were some significant yet small gender differences in emotion controllability beliefs in studies with a focus on specific aspects of emotion self-efficacy. Caprara et al. (2010) found that boys had stronger self-efficacy to manage negative affect; Mesurado et al. (2018) found boys had higher self-efficacy to manage despondency and distress, and Perchtold et al. (2019) found that males had higher self-efficacy in managing negative emotions.

## Discussion

A growing body of evidence indicates that emotion controllability beliefs influence both the likelihood a young person will attempt to regulate their emotions, and their subsequent mental health. Yet, to date, no review of this work has been undertaken. The current review examined emotion controllability beliefs in young people and how these beliefs are associated with symptoms of anxiety and depression, as well as the role that emotion regulation plays in these links. Taken as a whole, the findings of the reviewed studies indicate that emotion controllability beliefs matter for both anxiety and depression, across genders, and across a wide age range. Effect sizes ranged from small to large, with the studies of personal emotion controllability beliefs typically having higher effect sizes than those using general emotion controllability beliefs measures.

The reviewed studies consistently supported theoretical models (Ford & Gross, 2019, Kneeland, Dovidio, et al., 2016) linking emotion controllability beliefs with anxiety and depression symptoms via emotion regulation strategies that target emotional experience, such as cognitive reappraisal. In contrast, and also consistent with theory, there was little support for suppression as a mediator. Tamir et al. (2007) and others (e.g., Ford & Gross 2019) argue this is because when one is asked about emotion, it calls to mind emotional experience, rather than expressive behavior and therefore one would expect emotion controllability beliefs to be linked with emotion regulation strategies that target emotional experience (such as cognitive reappraisal), and not those that target emotional expression (such as suppression).

However, this explanation has not yet been directly tested. As the most widely used measures of emotion controllability beliefs are somewhat ambiguous in terms of whether they are asking respondents about controlling the experience or expression of emotion, further work on assessing emotion controllability beliefs is needed to fully understand the nature of these beliefs and how they relate to different emotion channels (emotional experience versus emotional expression).

Kneeland et al. (2016) also point out that motivation to regulate emotions, underpinned by the belief that emotions are relatively controllable, will only be impactful if an individual also has the ability to use a particular emotion regulation strategy (or indeed to regulate social interactions). At the same time, motivation to engage in a strategy because of a belief that emotions are controllable may be a necessary precursor to developing emotion regulation skills. Conceptual models suggest that beliefs that emotions are controllable versus uncontrollable may set young people on healthy versus unhealthy trajectories. A healthy trajectory could involve a young person being motivated to regulate their emotions, subsequently practicing effective emotion regulation strategies, and as a result experiencing positive mental health benefits. This would further confirm their beliefs, encouraging repeated engagement in emotion regulation strategies and the development of a wider repertoire of emotion regulation skills over time. An unhealthy trajectory could involve a young person being less motivated to engage in and practice emotion regulation strategies and subsequently only develop a limited range of emotion regulation skills, which could exacerbate the impact of challenging experiences on symptoms of anxiety and depression.

### Contextual and Person-Related Factors

The large majority of studies in this review were carried out in Western contexts, with many of the participant samples made up of university students. Additionally, only three studies were carried out in low- and middle-income countries making it difficult to comment on these contexts with confidence. Evidence from studies with adult populations indicates that one’s beliefs are shaped by cultural values (Tamir & Gutentag, 2017), including those associated with income, and it is likely that culture also influences the out-comes of these beliefs.

Several authors raised the possibility that believing emotions are relatively controllable may weaken during adolescence. Indeed, there was a general trend across the studies indicating that younger participants were more likely to believe that emotions are controllable than older participants. However, this evidence is still limited, and further research needs to be carried out using samples with a wider age range and consistent measures.

Regarding differences in gender, the findings were largely mixed. Findings seemed to vary depending upon specific emotions or valence, with boys/men being more likely to hold beliefs that negative emotions are controllable and girls/women being more confident in their beliefs about controlling the expression of positive emotions. This gender difference could plausibly be explained by early gender socialization. For instance, girls are taught that emotions are “natural” for females and may thus subsequently believe they are not controllable, while boys are taught emotions should be controlled (e.g., “boys don’t cry”), internalizing a belief that they can be controlled (Barrett & Bliss-Moreau, 2009). It is also possible that there may be gender differences in dispositional precursors of emotion controllability beliefs, such as negative emotionality.

Other factors, such as psychiatric disorder or levels of distress also need to be further examined in relation to emotion controllability beliefs—only one study in this review included a sample with elevated levels of psychological distress. Future work needs to investigate how emotion controllability beliefs function within different clinical samples. Although evidence suggests that believing emotions are relatively controllable leads to better mental health outcomes, this may not be the case for all individuals. For example, those with Generalized Anxiety Disorder, may already be overly active in their attempts to control their emotions, and holding a view that emotions are controllable may in fact be detrimental for this population (Kneeland Dovidio et al., 2016).

### Limitations

The limitations of this systematic review should be noted. The review was based on a relatively small number of peer-reviewed studies that were largely cross-sectional or longitudinal in design. While the peer review process aims to increase research quality, it is possible that excluding non-peer reviewed and unpublished studies from the analysis may have introduced some bias in the outcomes. Furthermore, as stated above, there is minimal evidence of how emotion controllability beliefs relate to young people’s anxiety and depression symptoms in non-Western contexts. Although there were no restrictions regarding language of assessment or geographical location of studies, studies which were not reported in English were excluded. As a result, studies examining these constructs in non-English speaking contexts may have been missed.

### Future Directions and Implications for Practice

While the findings of this review are consistent with theoretical models stating that emotion controllability beliefs exert a causal influence on anxiety and depression symptoms, further intervention studies and studies that experimentally manipulate emotion controllability beliefs are needed to test these causal hypotheses more directly. Future research also needs to test more complex models. Several of the reviewed studies indicated that emotion controllability beliefs may be associated with depression symptoms via different mediators. There could also be an interpersonal dimension to the process, for example, believing emotions are relatively controllable may predict better interpersonal engagement and subsequently fewer symptoms of depression.

The influence of culture on emotion controllability beliefs is another area in need of further research. For this to happen, greater diversity across cultural contexts and study settings is required, including low- and middle-income country contexts. Several questions remain regarding the influence of age, gender, and levels of distress on emotion controllability beliefs. Further work in these areas may help us better understand links between emotion controllability beliefs and young people’s anxiety and depression symptoms. Finally, this review has highlighted the importance of having psychometric measures that can reliably measure emotion controllability beliefs and distinguish between emotional experience and emotional expression. The field would also benefit from measures which better examine valence-specific information, providing data on both negative and positive emotions.

Regarding implications for practice, there is consistent evidence to suggest that believing emotions are relatively uncontrollable may impair a young person’s ability to identify the need to regulate their emotions. Based on these findings, clinicians may want to consider incorporating a measure of emotion controllability beliefs in their early assessments to help identify optimal interventions for different individuals. For example, those who believe emotions cannot be controlled may benefit most from interventions targeting such beliefs, rather than a focus on regulatory strategies.

## Conclusion

The influence of emotion regulation on youth mental health is well established. This work has raised important questions regarding what leads young people to engage in emotion regulation. A growing body of evidence indicates that emotion controllability beliefs influence both emotion regulation and subsequent mental health. This review systematically examined this emergent body of literature. The review synthesized the evidence from cross-sectional, longitudinal, experimental, and qualitative studies of emotion controllability beliefs and examined associations with young people’s anxiety and depression symptoms. Consistent links between emotion controllability beliefs and symptoms of both anxiety and depression were found, with beliefs that emotions are relatively controllable being associated with fewer symptoms of anxiety and depression. The review also examined the mediating role of emotion regulation in these associations. The evidence suggests that believing emotions are controllable is associated with more adaptive emotion regulation (e.g., cognitive reappraisal), which in turn relates to fewer symptoms of anxiety and depression. Taken together, the review findings demonstrate that emotion controllability beliefs matter in youth emotion regulation, anxiety, and depression symptoms. Understanding emotion controllability beliefs is of prime importance for basic science and practice, as it will allow us to advance understanding of youth mental health and provide potential additional targets for managing symptoms of anxiety and depression in young people.

## Figures and Tables

**Table 1:** Age, gender, racial and/or ethnic identity, sample, and country of participants

Study	Age of participants	Gender of participants	Racial and/or ethnic identity of participants (as reported in original article)	Sample	Country of residence of participants
Caprara et al. (2008)	*M* = 18.7, 18.9, 19.5 *SD* = 0.9, 1.0, 1.5	1271 F, 1199 M	Italian sample: Almost all participants were of Italian extract. US sample: Non-Hispanic Caucasian 71%, Hispanic 10%, Other 5%. Bolivian sample: not reported.	University	Italy, USA, Bolivia
Caprara et al. (2010)	*M* = 12.81, *SD* = 0.77	227 F, 225 M	Not reported	High School	Italy
Caprara et al. (2013)	*M* = 20.86, *SD* = 0.88	225 F, 178 M	Study 1: All of Italian extract. Study 2: U.S. sample was non-Hispanic Caucasian (67%), Hispanic (10%), Asian (9%), and Native American (8%), with other groups accounting for less than 2%.	University/Community	Italy & USA
Crawford et al. (2021)	*M* = 12.03, *SD* = 2.38	381 F, 301 M	67.9% White, 11.3% African American, 9.1% Asian/Pacific Islander, 6.0% multiracial, 4.8% other, and 0.9% American Indian/Alaskan Native, 12.8% Latinx	Primary/Middle/High School	USA
Daniel et al. (2020)	*M* = 20.37, *SD* = 2.95	84 F, 29 M	Self-reported racial composition: 68.14% white, 7.08% African American/black, 15.93% Asian, 1.77% Middle Eastern, 7.08% multiple races; self-reported ethnicity: 2.65% Hispanic	University, Elevated anxiety	USA*
De Castella et al. (2013)	*M* = 19.1, *SD* = 1.6	145 F, 71 M	45% White Caucasian, 12% Chinese, 8% South/East Asian, 8% Hispanic, 8% African American, 6% Mixed, 5% Indian, 4% Mexican, and 3% Other	University	USA
Di Gunta et al. (2022)	*M = 15.56, SD = 0.77*	48 F, 55 M	Not reported	High School	Italy
Ford et al. (2018)	*M* = 15.5	83 F, 53 M	82% Caucasian, 7% African American, 2% Latino/Hispanic, 4% Asian/Island Pacific, and 5% other/multiracial participants	High school	USA
King & dela Rosa (2019)	*M* = 18.94, *SD* = 1.73	260 F, 95 M	100% Filipino	University	Philippines
Kneeland et al. (2016)	*M* = 21.6, *SD* = 4.19	59 F, 30 M	43 (48.3%) White, 10 (11.2%) African American/Black, 16 (18.0%) Asian or Pacific Islander, 6 (6.7%) Latino/His-panic, and 14 (15.7%) Multiracial	University	USA
Mesurado et al. (2018)	*M* = 14.70, *SD* = 0.68	225 F, 192 M	Not reported	High school	Spain
Niditch & Varela (2012)	*M* = 14.82, *SD* = 1.71	78 F, 46 M	Caucasian (n = 68), African American (n = 37), Latino (n = 11), Asian American (n = 6), or of other races or ethnicities (n = 2)	Middle/High school	USA
Perchtold et al. (2019)	*M*=22.42, *SD* = 3.15	67 F, 59 M	Not reported	University	Austria/Germany*
Russell et al. (2021)	*M* = 15.1, *SD* = 2.8	100 F, 47 M	Not reported	Community	Australia^+^
Schroder et al. (2015)	*M* = 19.42, *SD* = 1.31	284 F, 102 M	Study 1: European American (86.6%), African-American (5.7%), Biracial (3.1%), Asian (2.8%), Latino/Hispanic (2.6%), and Native American (0.5%). Study 2: Not reported.	University	USA
Schroder et al. (2019)	*M* = 18.07, *SD* = 0.33	236 F, 70 M	Not reported	University	USA
Skymba et al. (2020)	*M* = 16.17, *SD* = 1.16	316 F, 106 M	46.9% White, 29.6% Asian, 14.5% Latinx, 6.4% African American, and 2.6% other/multiracial	University, High school	USA
Tamir et al. (2007)	*M* = 18.22, *SD* = 0.66	245 F, 192 M	59% Caucasian, 29% Asian, 12% Latino, 5% African American, and 4% Native American	University	USA
Tang et al. (2022)	*M* = 16.8, *SD* = 0.36	7 F, 6 M	Not reported	High school	China
Weems et al. (2015)	*M* = 13.39, *SD* = 1.53	561 F, 497 M	91% African American, 6% ‘mixed African-American/other,’ 1% Hispanic, 1% Euro-American, 0.5% Asian, and 0.5% other	Disaster exposed, Primary/Middle/High School	USA
Zhu et al. (2021)	*M* = 20.65, *SD* = 1.34	1409 F, 326 M	Study 1: 100% Chinese. Study 2: 94.8% Chinese, 2.5% non-Chinese, 2.7% did not identify	University	China

In age column, M = mean, SD = standard deviation. In gender column F = female, M = male

* Indicates country of university or first author affiliation when no other information on country of participants was provided

+ Also included recruitment via social media in other English-speaking countries: UK, USA, Canada, NZ

**Table 2:** Quality assessment of quantitative studies

Quality criteria for quantitative studies													
Study	Objective	Design	Select/ Variables	Subject	Out-come	Sample size	Analysis	Variance	Con-founds	Results	Conclusions	Quality Score (Max. 1)	Relevance
Caprara et al. (2008)	2	2	2	2	2	2	2	2	2	2	2	1.00	Medium
Caprara et al. (2010)	2	2	2	2	2	2	2	2	2	2	2	1.00	High
Caprara et al. (2013)	2	1	2	2	2	2	2	2	2	2	2	0.96	Medium
Crawford et al. (2021)	2	2	2	2	2	2	2	2	2	2	2	1.00	Medium
Daniel et al. (2020)	2	1	2	2	1	1	1	2	2	2	2	0.82	Medium
Di Gunta et al. (2022)	2	2	2	2	2	2	2	2	2	2	2	1.00	Medium
De Castella et al. (2013)	2	1	2	2	2	2	2	2	2	2	2	0.95	High
Ford et al. (2018)	2	2	2	2	2	2	2	2	2	2	2	1.00	High
King & dela Rosa (2019)	2	1	1	1	1	2	2	2	2	2	2	0.82	High
Kneeland et al. (2016)	2	2	2	2	2	2	2	2	2	2	2	1.00	High
Mesurado et al. (2018)	2	2	2	2	2	2	2	2	2	2	1	0.95	Low
Niditch & Varela (2012)	2	1	2	2	2	1	2	2	1	2	2	0.86	Medium
Perchtold et al. (2019)	1	1	2	1	2	2	2	2	2	2	2	0.86	High
Russell et al. (2021)	2	1	2	1	2	2	2	1	2	2	2	0.86	High
Schroder et al. (2015)	1	2	1	2	2	2	2	2	2	2	2	0.95	High
Schroder et al. (2019)	2	1	1	1	2	2	2	2	2	2	2	0.86	Medium
Skymba et al. (2020)	2	1	2	2	2	1	2	2	2	2	2	0.90	High
Tamir et al. (2007)	2	2	2	2	2	2	2	2	2	2	2	1.00	High
Weems et al. (2015)	2	1	2	2	2	2	2	2	1	1	1	0.82	Medium
Zhu et al. (2021)	1	2	1	2	2	2	2	2	1	1	1	0.77	Low

0 = not meeting, 1 = partially meeting, 2 = fully meeting criteria

Quality Criteria: *Objective* = clear description of research question/objective; *Design* = clear and appropriate (cross-sectional = 1); *Select/Variables* = selection of participants and independent variables sufficiently described and appropriate; *Subject* = subject characteristics sufficiently described; *Outcome* = outcome/any exposure measure(s) defined and robust to measurement bias/means of assessment reported; *Sample size* = sample size appropriate; *Analysis* = analytic methods described, justified and appropriate; *Variance* = estimate of variance reported for main results; *Confounds* = controlled for confounding variables; *Results* = results reported in sufficient detail; *Conclusions* = conclusions supported by results. *Quality Score* = all scores divided by the maximum score of 22. *Relevance* = usefulness of the study in addressing the research questions for this review

**Table 3:** Quality assessment of qualitative studies

Quality criteria for qualitative studies												
Study	Question	Design	Context	Theory	Sampling	Methods	Analysis	Verification	Conclusion	Reflexivity	Quality Score (Max. 1)	Relevance
Tang et al. (2022)	2	2	2	1	1	2	2	2	2	1	0.85	Medium

0 = not meeting, 1 = partially meeting, 2 = fully meeting criteria

Quality Criteria: *Question* = sufficiently described question/objective; *Design* = clear and appropriate; *Context* = clear context for study; *Theory* = connection to a theoretical framework/wider body of knowledge; *Sampling* = sampling strategy described, relevant and justified; *Methods* = data collection methods clearly described and systematic; *Analysis* = analytic methods clearly described and systematic; *Verification* = use of verification procedure(s) to establish credibility; *Conclusions* = conclusions supported by results; *Reflexivity* = reflexivity of the account. *Quality Score* = all scores divided by the maximum score of 22. *Relevance* = usefulness of the study in addressing the research questions for this review

**Table 4:** Overview of studies and key findings Studies using a general measure of emotion controllability beliefs

Studies using a general measure of emotion controllability beliefs							
Study	Design		ECB Measure	Anxiety	Depression	Key findings	Mediators
Crawford et al. (2021)	Longitudinal		ITES	–	✓	Believing emotions are controllable was negatively associated with negative emotionality Believing emotions are controllable was associated with less negative emotionality	Indirect effect of negative emotionality on depressive symptoms through ECBs
Ford et al. (2018) (Study 1)	Cross-sectional		ITES (adapted wording for a younger sample)	–	✓	Believing emotions are controllable was associated with fewer depressive symptoms	Indirect effect of ECBs on depressive symptoms through reappraisal (but not suppression)
King & dela Rosa (2019)	Cross-sectional		ITES using second person phrasing	✓	✓	Believing emotions are controllable was associated with lower levels of negative emotions, anxiety, and depression Believing emotions are controllable was associated with higher levels of life satisfaction and positive emotions Reappraisal was not associated with negative emotions, depression or anxiety	Indirect effect of ECBs on well-being (but not anxiety or depression) through cognitive reappraisal
Kneeland et al. (2016) *	Experimental		ITES Coping Self Efficacy Scale (Chesney et al. 2006)	✓	–	Participants who were induced to believe emotions are relatively controllable were more likely to use reappraisal in a subsequent negative mood induction, but were not more (or less) likely to use suppression Reappraisal was not associated with a decrease in negative affect or anxiety	N/A
Skymba et al. (2020)	Cross-sectional		8-item novel emotion mindset measure - adapted from ITES and EMS (Livingstone. 2012)	–	✓	Believing emotions are controllable was associated with more voluntary engagement and less disengagement and emotion dysregulation Higher voluntary engagement was associated with lower depressive symptoms Higher disengagement and emotion dysregulation was associated with higher depressive symptoms	Indirect effect of ECBs on depressive symptoms through voluntary engagement, disengagement, and emotion dysregulation
Tamir et al. (2007) *	Longitudinal		ITES Emotion regulation self-efficacy	–	✓	Believing emotions are controllable was associated with higher emotion regulation self-efficacy and cognitive reappraisal Believing emotions are controllable was associated with higher well-being, lower depressive symptoms, and better social adjustment one year later	Indirect effect of General ECBs on emotional (but not social) outcomes through Personal ECBs
Studies using a personal	measure of emotion controllability beliefs						
Study	Design		ECB Measure	Anxiety	Depression	Key findings	Mediators considered
Caprara et al. (2008)	Cross-sectional		Regulatory Emotional Self Efficacy (RESE), 14 items (Bandura et al.. 2003; Caprara & Gerbino. 2001)	–	✓	In the Italian sample, all three dimensions of the RESE scale were negatively associated with a youth self-report scale of anxiety and depression	N/A
Caprara et al. (2010)	Longitudinal		Regulatory Emotional Self-efficacy Scale, 12 items (Caprara & Gerbino. 2001; Caprara. Di Giunta. Eisenberg et al.. 2008)	–	✓	Perceived capacity to manage negative emotions and express positive emotions was associated with depression and delinquency concurrently and longitudinally Earlier self-regulation problems and exposure to family violence predicted beliefs that emotions were relatively uncontrollable	Indirect effect of self-efficacy to manage negative emotions on depression through interpersonal social self-efficacy
Caprara et al. (2013)	Cross-sectional		Multidimensional Negative Emotions Self-Regulatory Efficacy Scale (Caprara et al.. 2013)	–	✓	Self-efficacy beliefs regarding the management of despondency/sadness negatively predicted depression	N/A
Daniel et al. (2020)	Cross-sectional		ITES using first person phrasing	✓	✓	Believing emotions are controllable was associated with more positive (vs. negative) affect, after controlling for social anxiety symptom severity ECBs unrelated to daily use of emotion regulation strategies (including cognitive reappraisal and 18 other strategies)	N/A
De Castella et al. (2013) *	Cross-sectional		ITES (original items) ITES using first person phrasing	–	✓	Believing emotions are controllable was associated with higher well-being and decreased depression Personal ECBs explained greater unique variance than General ECBs	Indirect effect of ECBs on depression through reappraisal
Di Gunta et al. (2022)	Longitudinal (though ECB and depressive symptoms assessed concurrently)		Regulatory Emotional Self Efficacy (RESE), and mEMA protocol	–	✓	Maternal rejection was associated with higher depressive symptoms Higher depressive symptoms were associated with lower self-efficacy in dealing with sadness and higher dysregulation of both anger and sadness	Indirect effect of maternal rejection on depressive symptoms through ECBs
Mesurado et al. (2018)	Longitudinal		Regulatory Emotional Self Efficacy (RESE). 14 items (Bandura et al.. 2003; Caprara & Gerbino. 2001)	✓	✓	An indirect effect of depression on prosocial behaviour through perceived self-efficacy in expressing positive affect (but not negative affect) An indirect effect of depression on aggression through perceived self-efficacy in expressing positive affect (but not negative affect)	See key findings column
Niditch & Varela (2012)	Cross-sectional		Emotional self-efficacy scale of the Self-Efficacy Questionnaire for Children (Muris 2001)	✓	–	Emotional self-efficacy and maternal rejection predicted anxiety Maternal rejection (but not paternal rejection or control by either parent) predicted emotional self-efficacy	Indirect effect of maternal rejection on anxiety through emotional self-efficacy
Perchtold et al. (2019)	Cross-sectional		Emotion regulation subscale of the Self-report Emotional Ability Scale	✓	✓	Higher self-efficacy in managing negative emotions in men than in women Higher capacity for reappraisal generation predicted fewer depressive symptoms in men only	N/A
Russell et al. (2021)	Cross-sectional		Self-efficacy for emotion regulation (SE-ER)	✓	✓	When added into a hierarchical multiple regression, self-efficacy for emotion regulation explained an additional 11% of variance in anxiety and 34% variance in depression Self-efficacy for emotion regulation was a stronger predictor than all others: age, gender, positive/negative meta worry, monitoring, physical function, cognitive monitoring	N/A
Tang et al. (2022)	Cross-sectional Qualitative		Interview questions	–	✓	One key theme related to participants’ beliefs that certain features of emotions cannot be regulated	N/A
Studies using anxiety-and depression-specific measures of emotion controllability beliefs							
Study	Design		ECB Measure	Anxiety	Depression	Key findings	Mediators considered
Schroder et al. (2015)	Cross-sectional		ITES Implicit Theories of Anxiety Scale (TOA)―using second person phrasing	✓	✓	General ECBs and anxiety-specific ECBs both independently predicted anxiety and depression Believing emotions are controllable was associated with more reappraisal and less suppression Individuals who believed emotions are relatively controllable were more likely to choose individual therapy over medication	N/A
Schroder et al. (2019)	Longitudinal		ITES TOA―using second person phrasing	✓	✓	Anxiety-specific ECBS predicted future weekly distress (a composite of worry, anxiety, loneliness, depression, and anger items), after controlling for the previous week’s distress, sex, socioeconomic status, baseline depression symptoms, and presence of psychiatric diagnosis	N/A
Weems et al. (2015)		Cross-sectional	Anxiety Control Questionnaire – Short version (Weems. 2005)	✓	–	Anxiety-specific ECBS associated with anxiety symptoms beyond level of exposure to hurricanes in youth	N/A
Zhu et al. (2021)		Cross-sectional	Mindsets of Depression, Anxiety, and Stress (MDASS) Added depression items to TOA. Uses second person phrasing in some scales. Chinese language	✓	✓	Believing emotions are controllable was associated with fewer depression, anxiety, and stress symptoms	N/A

*ECBs* emotion controllability beliefs, *ITES* Implicit Theories of Emotion Scale (Tamir et al., 2007), *mEMA* mobile ecological momentary assessment

* This study used both a personal and a general measure of emotion controllability; – = not assessed

## References

[R1] Aldao A, Nolen-Hoeksema S, Schweizer S (2010). Emotion-regulation strategies across psychopathology: A meta-analytic review. Clinical Psychology Review.

[R2] Bandura A (1997). Self-efficacy: The exercise of control.

[R3] Bandura A, Caprara GV, Barbaranelli C, Gerbino M, Pastorelli C (2003). Role of affective self-regulatory efficacy on diverse spheres of psychosocial functioning. Child Development.

[R4] Barrett LF, Bliss-Moreau E (2009). She’s emotional. He’s having a bad day: Attributional explanations for emotion stereotypes. Emotion.

[R5] Becerra R, Preece DA, Gross JJ (2020). Assessing beliefs about emotions: Development and validation of the emotion beliefs Questionnaire. PLOS ONE.

[*] Caprara GV, Di Giunta L, Eisenberg N, Gerbino M, Pastorelli C, Tramontano C (2008). Assessing regulatory emotional self-efficacy in three countries. Psychological Assessment.

[R7] Caprara GV, Gerbino M, Caprara GV (2001). Self-efficacy assessment.

[*] Caprara GV, Gerbino M, Paciello M, Di Giunta L, Pastorelli C (2010). Counteracting depression and delinquency in late adolescence: The role of regulatory emotional and interpersonal self-efficacy beliefs. European Psychologist.

[*] Caprara GV, Di Giunta L, Pastorelli C, Eisenberg N (2013). Mastery of negative affect: A hierarchical model of emotional self-efficacy beliefs. Psychological Assessment.

[R10] Chesney MA, Neilands TB, Chambers DB, Taylor JM, Folkman S (2006). A validity and reliability study of the coping self-efficacy scale. British Journal of Health Psychology.

[*] Crawford CM, Griffith JM, Hankin BL, Young JF (2021). Implicit beliefs about emotions in youth: Associations with temperamental negative emotionality and depression. Journal of Social and Clinical Psychology.

[*] Daniel KE, Goodman FR, Beltzer ML, Daros AR, Boukhechba M, Barnes LE, Teachman BA (2020). Emotion malleability beliefs and emotion experience and regulation in the daily lives of people with high trait social anxiety. Cognitive Therapy and Research.

[*] De Castella K, Goldin P, Jazaieri H, Ziv M, Dweck CS, Gross JJ (2013). Beliefs about emotion: Links to emotion regulation, well-being, and psychological distress. Basic and Applied Social Psychology.

[*] Di Giunta L, Lunetti C, Gliozzo G, Rothenberg WA, Lansford JE, Eisenberg N, Pastorelli C, Basili E, Fiasconaro I, Thartori E, Favini A (2022). Negative parenting, adolescents’ emotion regulation, self-efficacy in emotion regulation, and psychological adjustment. International Journal of Environmental Research and Public Health.

[R15] Diener CI, Dweck CS (1978). An analysis of learned helplessness: Continuous changes in performance, strategy, and achievement cognitions following failure. Journal of Personality and Social Psychology.

[R16] Dweck CS (2006). Mindset: The new psychology of success.

[R17] Ford BQ, Gross JJ (2019). Why beliefs about emotion matter: An emotion-regulation perspective. Current Directions in Psychological Science.

[*] Ford BQ, Lwi SJ, Gentzler AL, Hankin B, Mauss IB (2018). The cost of believing emotions are uncontrollable: Youths’ beliefs about emotion predict emotion regulation and depressive symptoms. Journal of Experimental Psychology: General.

[R19] Gough D, Oliver S, Thomas J (2017). An introduction to systematic reviews.

[R20] Gross JJ, John OP (2003). Individual differences in two emotion regulation processes: Implications for affect, relationships, and well-being. Journal of Personality and Social Psychology.

[*] King RB, dela Rosa ED (2019). Are your emotions under your control or not? Implicit theories of emotion predict well-being via cognitive reappraisal. Personality and Individual Differences.

[R22] Kmet LM, Cook LS, Lee C (2004). Standard quality assessment criteria for evaluating primary research papers from a variety of fields. Alberta Heritage Foundation for Medical Research.

[R23] Kneeland ET, Dovidio JF, Joormann J, Clark MS (2016). Emotion malleability beliefs, emotion regulation, and psychopathology: Integrating affective and clinical science. Clinical Psychology Review.

[*] Kneeland ET, Nolen-Hoeksema S, Dovidio JF, Gruber J (2016). Emotion malleability beliefs influence the spontaneous regulation of social anxiety. Cognitive Therapy and Research.

[R25] Livingstone KM (2012). The effects of implicit theories of emotion on emotion regulation and experience.

[*] Mesurado B, Vidal EM, Mestre AL (2018). Negative emotions and behaviour: The role of regulatory emotional self-efficacy. Journal of Adolescence.

[R27] Moltrecht B, Deighton J, Patalay P, Edbrooke-Childs J (2021). Effectiveness of current psychological interventions to improve emotion regulation in youth: A meta-analysis. European Child & Adolescent Psychiatry.

[R28] Muris P (2001). A brief questionnaire for measuring self-efficacy in youths. Journal of Psychopathology and Behavioral Assessment.

[*] Niditch LA, Varela RE (2012). Perceptions of parenting, emotional self-efficacy, and anxiety in youth: Test of a mediational model. Child & Youth Care Forum.

[*] Perchtold CM, Papousek I, Fink A, Weber H, Rominger C, Weiss EM (2019). Gender differences in generating cognitive reappraisals for threatening situations: Reappraisal capacity shields against depressive symptoms in men, but not women. Frontiers in Psychology.

[*] Russell JK, Strodl E, Kavanagh DJ (2021). Correlates of distress in young people with cystic fibrosis: The role of self-efficacy and metacognitive beliefs. Psychology & Health.

[R32] Schaefer J, Naumann E, Holmes E, Tuschen-Caffier B, Samson A (2017). Emotion regulation strategies in depressive and anxiety symptoms in youth: A meta-analytic review. Journal of Youth and Adolescence.

[*] Schroder HS, Dawood S, Yalch MM, Donnellan MB, Moser JS (2015). The role of implicit theories in mental health symptoms, emotion regulation, and hypothetical treatment choices in college students. Cognitive Therapy and Research.

[*] Schroder HS, Callahan CP, Gornik AE, Moser JS (2019). The fixed mindset of anxiety predicts future distress: A longitudinal study. Behavior Therapy.

[*] Skymba HV, Troop-Gordon W, Modi HH, Davis MM, Weldon AL, Xia Y, Heller W, Rudolph KD (2020). Emotion mind-sets and depressive symptoms in adolescence: The role of emotion regulation competence. Emotion.

[*] Tamir M, Gutentag T (2017). Desired emotional states: Their nature, causes, and implications for emotion regulation. Current Opinion in Psychology.

[R37] Tamir M, John OP, Srivastava S, Gross JJ (2007). Implicit theories of emotion: Affective and social outcomes across a major life transition. Journal of Personality and Social Psychology.

[R38] Tang X, Wong DFK, Xu H, Hou L (2022). Barriers to a class-room-based universal prevention program for depressive symptoms in chinese adolescents: A qualitative study. Health & Social Care in the Community.

[R39] Weems CF (2005). The anxiety control questionnaire for children-short form.

[*] Weems CF, Russell JD, Graham RA, Neill EL, Banks DM (2015). Developmental differences in the linkages between anxiety control beliefs and posttraumatic stress in youth. Depression and Anxiety.

[*] Zhu S, Zhuang Y, Lee P (2021). Psychometric properties of the Mindsets of Depression, anxiety, and stress scale (MDASS) in chinese young adults and adolescents. Early Intervention in Psychiatry.

